# Collection and Curation of Transcriptional Regulatory Interactions in *Aspergillus nidulans* and *Neurospora crassa* Reveal Structural and Evolutionary Features of the Regulatory Networks

**DOI:** 10.3389/fmicb.2018.00027

**Published:** 2018-01-19

**Authors:** Yibo Hu, Yuqi Qin, Guodong Liu

**Affiliations:** ^1^State Key Laboratory of Microbial Technology, School of Life Science, Shandong University, Jinan, China; ^2^Hunan Provincial Key Laboratory of Microbial Molecular Biology, State Key Laboratory of Developmental Biology of Freshwater Fish, College of Life Science, Hunan Normal University, Changsha, China; ^3^National Glycoengineering Research Center, Shandong University, Jinan, China

**Keywords:** transcription factor, transcriptional regulatory network, filamentous fungi, *Aspergillus nidulans*, *Neurospora crassa*

## Abstract

Transcriptional regulation has important roles in various biological processes (e.g., development and metabolism) in filamentous fungi. However, regulatory interactions between transcription factors (TFs) and their target genes in these species have only been described in different forms by primary scientific literature, which limits the integrated analysis of these data. Here, we extensively curated the reported transcriptional regulatory interactions in *Aspergillus nidulans* and *Neurospora crassa*. For each interaction, the identifiers of involved proteins or genes were unified, and the types of supporting experiments were recorded. Then, transcriptional regulatory networks were reconstructed from the interactions supported by classical low-throughput experiments. Analysis of the networks revealed the presence of hub targets regulated by multiple TFs and network motifs of other structures (e.g., regulatory loops). Comparison of the regulatory interactions between the two species identified 33 conserved interactions supported by classical experiments in both species, most of which are involved in the regulation of metabolic genes. We anticipate the curated data would serve as a catalog for the studies of transcriptional regulation in filamentous fungi.

## Introduction

Investigation of the regulatory interactions between transcription factors (TFs) and their targets is important for the understanding and engineering of biological processes. Genome-scale reconstruction of transcriptional regulatory networks, which contain TFs, target genes, as well as their interactions, has been one of the key tasks in systems biology (Herrgård et al., 2004). Different from that of metabolic networks, the reconstruction of transcriptional regulatory networks is largely dependent on experimental approaches due to the limited conservation of TF–target relationships between different species (Feist et al., 2009). For this reason, global transcriptional regulatory networks have only been constructed in some model organisms, with related databases (e.g., HTRIdb, Bovolenta et al., 2012; RegulonDB, Gama-Castro et al., 2016; and YEASTRACT, Teixeira et al., 2014) developed as repositories of experimentally clarified regulatory interactions. The structural and evolutionary characteristics of the networks have been studied in some species (Guzman-Vargas and Santillan, 2008; Seo et al., 2015). When being integrated with metabolic networks, transcriptional regulatory networks were successfully used for the modeling of cell behaviors (Chandrasekaran and Price, 2010).

Filamentous fungi have attracted significant research interests for their important roles in human life (e.g., as plant pathogens or antibiotic producers) and basic research of eukaryotic cells (e.g., cell differentiation and communication) (Timberlake and Marshall, 1989). With the increasing availability of fungal genome sequences, it is now possible to predict the number and types of TFs in many fungi using computational methods (Park et al., 2008), and their evolutionary diversity across species has been systematically analyzed (Todd et al., 2014). However, the regulatory relationships between TFs and their targets in filamentous fungi have been only dispersedly reported in different formats. For example, one regulatory interaction could be independently reported by different research groups using different experimental methods and even giving different names to the involved genes. Therefore, extraction of these fragmented information from scientific literature and subsequent data organization and integration would be helpful to understand transcriptional regulations at the systems level. Ideally, TF–target interaction data in model as well as economically or medically important fungal species could be curated and deposited in a database for reuse and analysis. However, to our knowledge, currently an extensive catalog of TF–target interactions is not available in filamentous fungi.

*Aspergillus nidulans* and *Neurospora crassa* have been used as the main models to study various biological processes including conidiation (Park and Yu, 2012), cell fusion (Fleissner et al., 2008), DNA damage response (Goldman and Kafer, 2004), circadian rhythm and lignocellulose degradation (Roche et al., 2014), during which some small-scale transcriptional regulatory networks have been constructed (Tudzynski, 2014; Zámborszky et al., 2014). In this study, we manually collected and curated the documented interactions between TFs and targets in these two model filamentous fungi. The types of interactions and related experimental methods were recorded in standard electronic formats. Then, TF–target interactions supported by classical low-throughput studies were used for the reconstruction and analysis of transcriptional regulatory networks.

## Materials and methods

### Curation of TF–target interactions

To collect research papers containing information on TF–target interactions, we searched articles in PubMed with the queries “(nidulans[Title/Abstract]) AND transcription [Title/Abstract],” “(crassa[Title/Abstract]) AND transcription [Title/Abstract]” and their variants (e.g., replacing “transcription” by “transcriptional”). In addition, we retrieved the lists of predicted TFs in the two species from FTFD, a database computationally predicting fungal TFs at genome scale (Park et al., 2008). For *A. nidulans*, the curated papers associated with each TF in the database AspGD (Cerqueira et al., 2014) were also collected. For *N. crassa*, we directly searched articles in PubMed with the names of characterized TFs. In total, more than 1,000 papers were collected and checked for the presence of information on TF–target interactions. It should be noticed that new research papers providing TF–target interactions (e.g., Virgilio et al., 2017) have been continuously published during the preparation of this manuscript, which need to be collected in the future.

**Figure 1:**
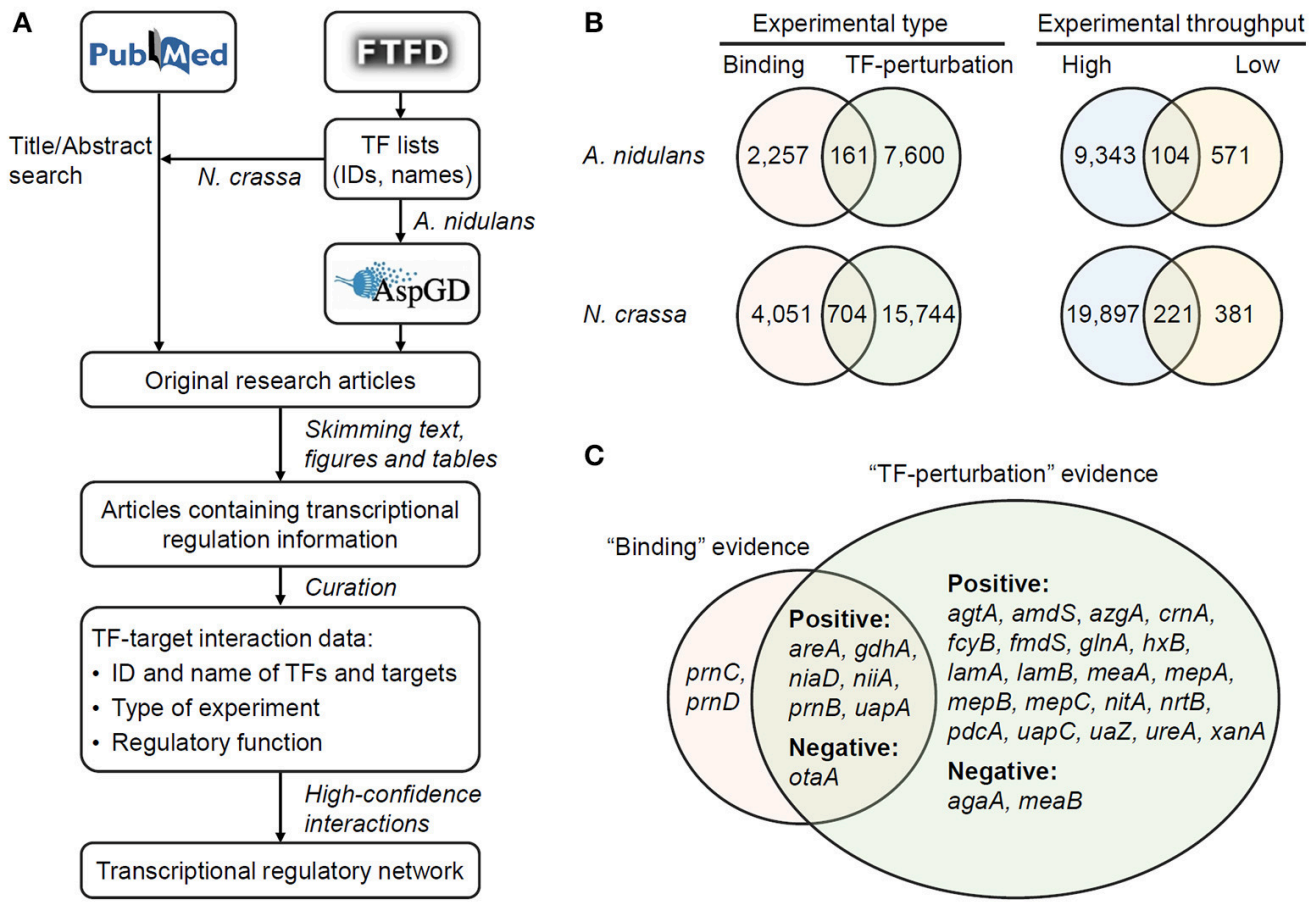
Collection and curation of transcriptional regulatory interactions. **(A)** Workflow of the study. **(B)** The number of TF–target interactions evidenced by different types and throughputs of experiments. **(C)** The targets of AreA in *A. nidulans* supported by different experimental types collected from 45 original research articles. The targets regulated in positive- (i.e., activated by AreA) and negative- (i.e., repressed by AreA) manners are indicated.

For each regulatory interaction, the ID and name (if applicable) of TFs and targets, the type of experimental evidence, the regulatory function (repression or activation), and the PubMed ID of reference literature were recorded. Perturbations of TFs through gene deletion, knock-down of gene expression, protein inactivation, or similar manners were recorded as “TFdown” manipulations, while those through gene overexpression or introduction of gain-of-function mutants were classified into “TFup” manipulations. The names and IDs of proteins were completed and occasionally corrected according to the protein sequences and annotations in AspGD (*A. nidulans* version s10-m04-r06) and BROAD ftp site (*N. crassa* version 12, http://www.broadinstitute.org/ftp/pub/annotation/fungi/neurospora_crassa/). Protein IDs in *N. crassa* were also linked to the accession numbers in GenBank (BioProject PRJNA132). In TF-perturbation studies, the gene encoding the perturbed TF is often included in the differentially-expressed gene set in transcriptome data, but should not be considered as the target of its product. The cultivation conditions and strengths of regulatory interactions, although important for the understanding of transcriptional regulations (Geistlinger et al., 2013), were not curated due to the difficulty in developing standard formats. The process of data collection and curation was summarized in Figure 1A.

### Visualization and analysis of networks

For the curated regulatory interactions supported by low-throughput experiments, the IDs of TFs, their targets, and the type of supporting experiments (“Binding,” “TF-perturbation,” or “Both”) were saved in a tab-delimited text file. The file was imported to Cytoscape 3.5.1 (Shannon et al., 2003), where the columns containing TF IDs, target IDs and experimental types were selected. The Cytoscape built-in plugin NetworkAnalyzer (Assenov et al., 2008) was subsequently applied to calculate the parameters of the networks (treated as directed edges) using default settings. The average number of neighbors was shown in the tab “Simple Network Parameters” of the analysis result.

### Identification of orthologous regulations

Putative orthologous proteins between pairs of fungal species were acquired from the Fungal Orthogroups Repository (https://portals.broadinstitute.org/regev/orthogroups/). The *Saccharomyces cerevisiae* protein IDs were searched in the YEASTRACT database (Teixeira et al., 2014) to check if there are documented regulatory relationships using the “Search for Associations” tool with default parameters.

## Results and discussion

### Curation of transcriptional regulatory interactions

Our manual curation of TF–target interactions resulted in data associated with 81 and 66 TFs in *A. nidulans* and *N. crassa*, respectively (Table 1 for basic statistics, all interactions listed in Table S1). Considering that a 608 and 459 TFs were predicted in the two species, respectively (Park et al., 2008), only a small fraction of TFs were covered. The result suggests our knowledge about the transcriptional regulatory networks in filamentous fungi lags far behind other model organisms like *S. cerevisiae*, in which almost all the TFs have been identified for their targets and about 163,000 regulatory interactions have been curated (Teixeira et al., 2014). Compared with the status in some “non-model” organisms such as pathogenic bacteria *Pseudomonas aeruginosa* (Galán-Vásquez et al., 2011) and *Mycobacterium tuberculosis* (Guo et al., 2009), the fractions of TFs known for targets in the two fungal species were also lower. Most of the interactions had one experimental evidence, while some others were supported by more than 10 records (Figure S1), revealing research preferences for specific regulatory interactions. As an example, regulation of alcohol dehydrogenase gene *alcA* by AlcR in *A. nidulans* was supported by 24 experiments from 20 papers, all published before the year 2005.

**Table 1:** Statistics of curated transcriptional regulatory interactions.

**Number**	***A. nidulans***	***N. crassa***
TFs	81	66
Target genes of TFs	5,944	7,442
Non-redundant regulatory interactions^a^	10,018	20,499
All regulatory interaction records^b^	10,867	22,402
Reference papers	294	158

a *Number of regulatory interactions between TFs and target genes regardless of the type of supporting experiments*.

b *Number of records if considering regulatory interactions supported by different experiments or difference literature as different records*.

The experimental evidences for the regulatory interactions were classified into two types. “Binding” evidences included those obtained with chromatin immuneprecipitation (ChIP), electrophoresis mobility shift assay (EMSA), DNA footprinting, and other methods detecting physical binding of TFs to target DNA. Some of the literature reported the exact binding sites of TFs, which were not covered in this study but are worth curating in the future. Interactions with “TF perturbation” evidences indicated those detected through the identification of differentially expressed genes after perturbing the abundance or activity of TFs. In total, 161 and 704 interactions were supported by both types of evidence in *A. nidulans* and *N. crassa*, respectively (Figure 1B). That is to say, these interactions involve direct binding of TFs to their target promoters which result in functional regulation of the transcription of the corresponding genes. For example, AreA, a nitrogen-responsive TF in *A. nidulans*, activates or represses seven of its targets by directly binding to their promoters (Figure 1C).

In addition, the experimental evidences were classified according to the throughput of methods. Microarray- or deep sequencing-based methods (e.g., ChIP-chip and RNA-seq) allow genome-wide mapping of the targets of TFs, while the confidences of identified targets are not necessarily high. In contrast, classical low-throughput studies (e.g., EMSA and Northern blot) only illustrate interactions between individual TFs and targets, but the information are generally of high confidence. Not surprisingly, only 6.7 and 2.9% of all the interactions had evidences from low-throughput studies in *A. nidulans* and *N. crassa*, respectively (Figure 1B). Particularly, a total of 20,118 interactions in *N. crassa* were supported by high-throughput experiments reported in just 27 reference papers.

### Structural features of transcriptional regulatory networks

**Figure 2:**
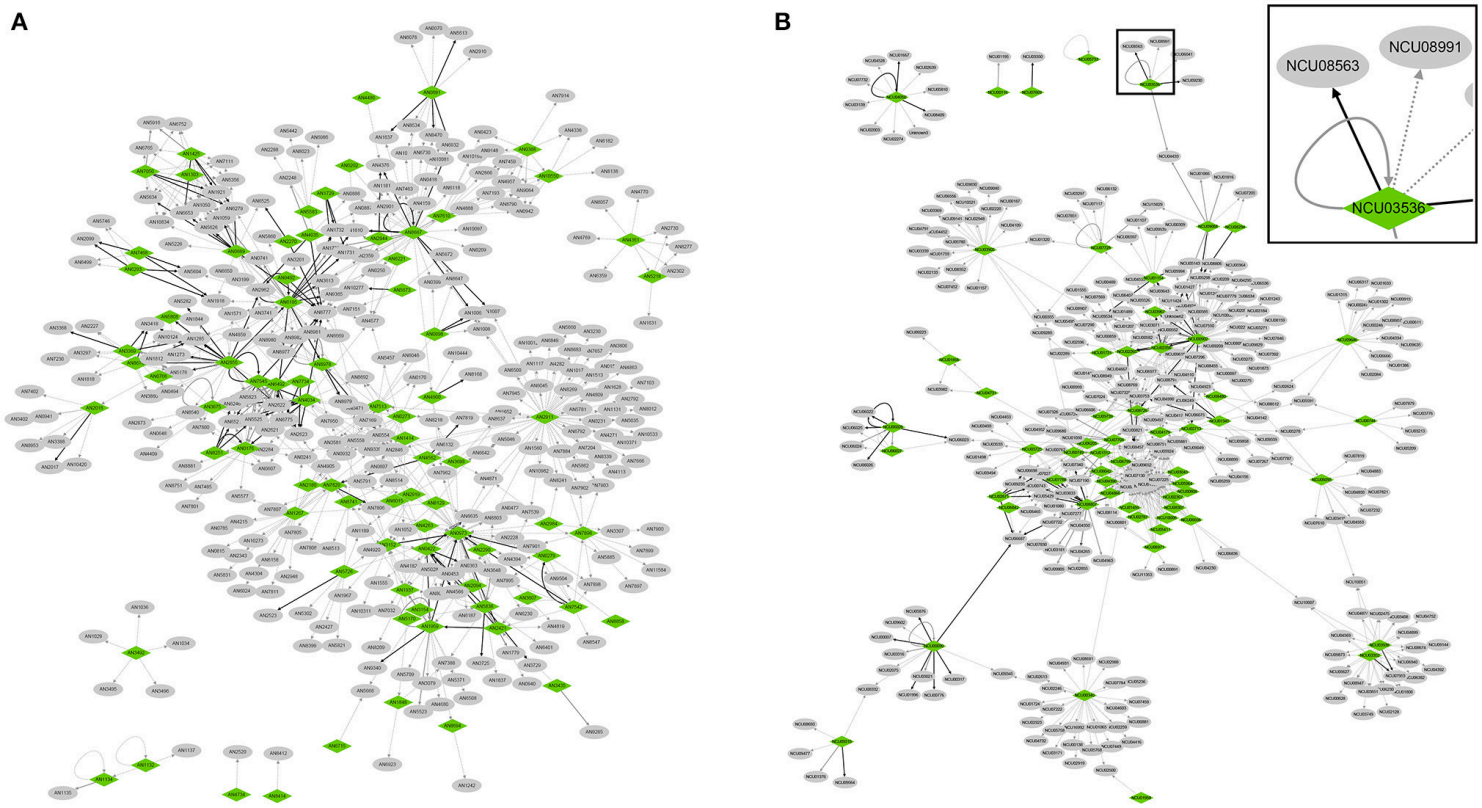
Visualization of transcriptional regulatory networks reconstructed in *A. nidulans* **(A)** and *N. crassa* **(B)**. An enlarged view of the boxed area in *N. crassa* network is shown at the top right corner. All the interactions are supported by low-throughput experiments. Nodes indicating TFs and non-TF targets are colored in green and gray, respectively. Regulatory interactions only supported by “Binding” experiments are indicated by gray solid arrows, while those only supported by “TF perturbation” experiments are indicated by gray dotted arrows. Interactions supported by both experiments are indicated by black solid arrows. The network images of high resolution are shown in Figure S2.

The regulatory interactions supported by low-throughput experiments were then used for the bottom-up reconstruction of transcriptional regulatory networks. The obtained networks of *A. nidulans* and *N. crassa* containing only high-confidence interactions were composed of 81 and 58 TFs, 675 and 602 TF–target interactions, respectively (Figure 2).

The networks recaptured some already established regulatory models. For example, a sub-network in *A. nidulans* clearly revealed the complex regulatory relationships between eight TFs involved in conidiation, with BrlA playing a central role in the regulation (Figure S3, see review by Park and Yu, 2012).

Both regulatory networks were well connected, with only six and five connected components (i.e., sub-network within which any two nodes are connected to each other by paths if considering the network as an undirected graph) detected in *A. nidulans* and *N. crassa*, respectively (Figure 2). The nodes in the networks had 3,462 and 3,451 neighbors in average, respectively, in the two species, as shown in the tab “Simple Network Parameters” of the result of NetworkAnalyzer. Specifically, we looked into regulatory interactions involved in four kinds of network motifs (i.e., small set of regulatory associations, Alon, 2007).

#### Combinatorial regulation

**Figure 3:**
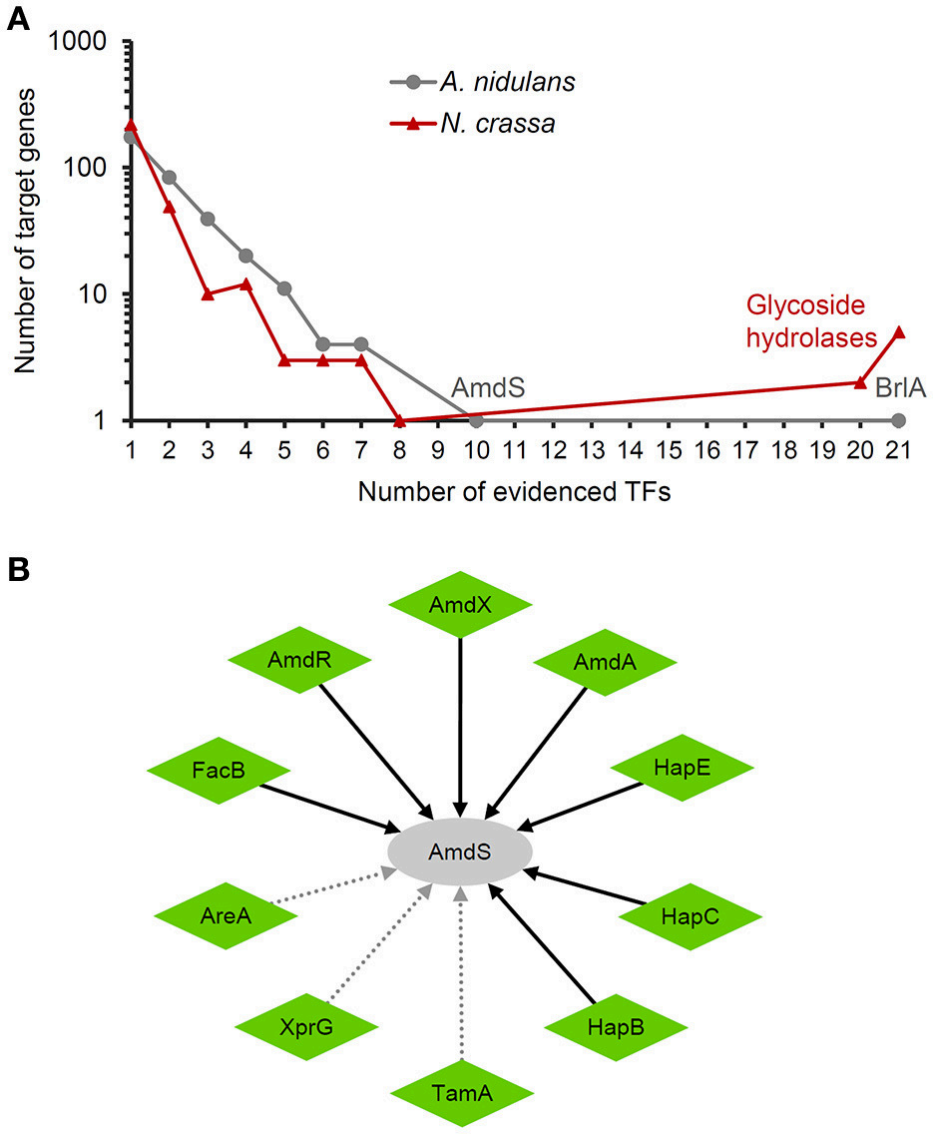
Combinatorial regulation of targets genes. All the interactions are supported by low-throughput experiments. **(A)** Distribution of the number of TFs per target gene. The two targets controlled by most TFs in *A. nidulans* are indicated. The seven targets controlled by most TFs in *N. crassa* encode glycoside hydrolases. **(B)** TFs regulating the expression of *amdS* in *A. nidulans*. The nodes and arrows are styled as indicated in the legend of Figure 2.

A significant feature of transcriptional regulation in eukaryotes is that gene expression is frequently regulated by combination of TFs (Lee et al., 2002). A comparison of the transcriptional regulatory networks in *S. cerevisiae* and *Escherichia coli* revealed that there were much more TF pairs regulating common genes in the former species (Guzman-Vargas and Santillan, 2008). Although the regulatory networks reconstructed here only covered a limited number of TFs, target genes regulated by multiple TFs were extensively observed (Figure 3A). In *A. nidulans*, 163 genes (48.4% of all target genes in the network) were regulated by two or more TFs. Particularly, *amdS* (gene ID: AN8777) encoding an acetamidase and *brlA* (AN0973) encoding a TF for conidiation in *A. nidulans* were regulated by 10 and 21 TFs, respectively, suggesting the tight control of the related biological processes by multiple environmental conditions. All the 10 TFs activated the expression of *amdS*, of which seven were proved to directly bind on its promoter (Figure 3B). Also, 88 genes (28.7% of all target genes in the network) were regulated by multiple TFs in *N. crassa*.

#### Autoregulation

Twelve and eight TFs were identified as autoregulating TFs in *A. nidulans* and *N. crassa*, respectively (Table S2). Specifically, in *A. nidulans*, AlcR (Lockington et al., 1987), AbaA, BrlA, QutA, PacC, StuA, and AreA activate, while QutR, CreA, and HapB repress, their own expression. Also, WC-1, CPC-1, ACR-2, QA-1F, and FL activate their own expression in *N. crassa*. These autoregulations might enhance or attenuate the regulatory outputs of TFs in response to environmental changes. In addition, VosA and CpcA in *A. nidulans* and PACC, CYS-3, SRE in *N. crassa* can bind to their own promoters, while the consequent regulatory effects have not been clarified through low-throughput experiments to our knowledge.

#### Regulatory cascades

We observed 93 and 46 regulatory interactions between TFs (i.e., regulation of a TF by another TF) in *A. nidulans* and *N. crassa*, respectively (Table S2). In total, 37 and 22 TFs can be regulated by other TFs, respectively. Although the strength and biological function of most of these regulatory cascades remain unknown, they might amplify, integrate, or sequentially transduce upstream signals in transcriptional regulation.

#### Bidirectional regulatory loops

Seven regulatory loops formed by bidirectional regulation of two TFs were observed in *A. nidulans* (Table S2), of which AbaA–BrlA, FlbB–FlbD, and FlbB–FlbD work as positive feedback loops and SreA–HapX works as a double-negative feedback loop. In *N. crassa*, nine of such TF pairs were observed, including WC-1–FRQ and WC-2–FRQ as positive feedback loops and CRE-1–COL-26 as a negative feedback loop. Such regulatory loops may result in bistable gene expression systems (Ferrell, 2002).

Since the networks used for analyzing the above types of regulatory associations only contained regulatory interactions supported by low-throughput experiments, we also used all the curated regulatory interactions regardless of the experiment type for network reconstruction. Investigation of this more comprehensive network confirmed the large number of combinatorial regulation, with 41.2 and 62.6% of all targets being regulated by more than one TF in *A. nidulans* and *N. crassa*, respectively. In addition, three more autoregulating TFs were identified in *N. crassa* (Table S2). Finally, 51 and 233 more regulatory interactions between TFs, some of which also formed bidirectional regulatory loops, were detected in *A. nidulans* and *N. crassa*, respectively (Table S2). Actually, TFs associated with autoregulation or regulatory cascades accounted for 84.0 and 95.5% of all the TFs in this study in the two species, respectively, highlighting the hierarchical structure of the transcriptional regulatory network in filamentous fungi.

### Conserved regulatory interactions between *A. nidulans* and *N. crassa*

Previous genome-scale analysis of the presence of TFs in fungi indicated that only a small part of them is evolutionarily conserved in the filamentous ascomycetes (Todd et al., 2014). For example, the above-mentioned AlcR and BrlA, which are well-studied in *A. nidulans*, do not have an ortholog in *N. crassa*. GalR, an activator of galactose utilizing genes, was only found in *A. nidulans* but no other fungi (Christensen et al., 2011). Further, even for orthologous TFs, their regulatory targets and consequent functions can vary largely between species (Lavoie et al., 2009). Here, the curated transcriptional regulatory interactions were compared between *A. nidulans* and *N. crassa* based on protein orthology.

A total of 5,842 putative orthologous relationships were identified between proteins in the two species. These include 27 pairs of TFs collected for targets in both species in this study. Of the 675 regulatory interactions supported by low-throughput experiments in *A. nidulans*, 371 had putative orthologs for both TF and target in *N. crassa*. However, only 40 interactions involving 18 TFs were evidenced by low-throughput experiments in both species (Table 2). Interestingly, most of the target genes associated to these shared interactions encode metabolic enzymes, including those in nitrogen metabolism, plant cell wall-polysaccharide degradation and cell redox homeostasis. This result could be linked to the relatively high conservation of metabolic pathways among biological processes (Peregrín-Alvarez et al., 2009). Some of these interactions were supported by different types of experiments in the two species, thus providing complementary knowledge for the regulations. For example, the TF ClrB in *A. nidulans* might directly binds to the promoter of the gene encoding the cellulase CbhB to activate its expression (Coradetti et al., 2012), as suggested by the interaction between CLR-2 and *cbh-1* (putative orthologs of ClrB and *cbhB*, respectively) in *N. crassa* (Craig et al., 2015).

**Table 2:** Conserved regulatory interactions in *A. nidulans* and *N. crassa* supported by low-throughput experiments in both species.

***A. nidulans***			***N. crassa***			**Function of target^b^**
**TF name**	**Target name**	**Experimental type^a^**	**TF name**	**Target name**	**Experimental type^a^**	
AcuK	*acuF*	Both	AOD-5	*acu-6*	P	Phosphoenolpyruvate carboxykinase
AcuK	*aodA*	Both	AOD-5	*aod-1*	Both	Alternative oxidase
AcuM	*acuF*	Both	AOD-2	*acu-6*	P	Phosphoenolpyruvate carboxykinase
AcuM	*aodA*	Both	AOD-2	*aod-1*	Both	Alternative oxidase
AreA	*nrtB*	P	NIT-2	*nit-10*	P	High-affinity nitrate transporter
AreA	*niaD*	Both	NIT-2	*nit-3*	Both	Nitrate reductase
AtfA	*catA*	P	ASL-1;ATF-1	*cat-1*	Both	Conidia-specific catalase
ClrA	*cbhD*	P	CLR-1	*gh6-2;cbh-2*	B	Cellulose 1,4-beta-cellobiosidase
ClrB	*cbhB*	P	CLR-2	*cbh-1*	Both	Cellulose 1,4-beta-cellobiosidase
ClrB	*cbhD*	P	CLR-2	*gh6-2;cbh-2*	B	Cellulose 1,4-beta-cellobiosidase
ClrB	*xlnC*	P	CLR-2	*gh10-1*	P	Endo-1,4-beta-xylanase
CpcA	*cpcA*	B	CPC-1	*cpc-1*	Both	Transcription factor of amino acid biosynthesis
CpcA	*argB*	P	CPC-1	*arg-12*	Both	Ornithine carbamoyltransferase
CreA	*xlnA*	Both	CRE-1	*gh11-1*	Both	Endo-1,4-beta-xylanase
FacB	*facA*	Both	ACU-15	*acu-5*	P	Acetyl-CoA synthase
FacB	*acuD*	Both	ACU-15	*acu-3*	P	Isocitrate lyase
FacB	*acuE*	Both	ACU-15	*acu-9*	P	Malate synthase
MetR	*sconC*	P	CYS-3	*scon-3*	P	Protein related to sulfur metabolism
MetR	*sconB*	P	CYS-3	*scon-2*	Both	Protein related to sulfur metabolism
NapA	*trxA;thiO*	P	NAP-1	*trx-1*	P	Thioredoxin
NapA	*sodA*	P	NAP-1	*sod-1*	P	Cu/Zn-superoxide dismutase
NapA	*glrA*	P	NAP-1	*glr-1*	P	Glutathione oxidoreductase
NapA	*gpxA*	P	NAP-1	*hyr-1;gpx-2*	P	Glutathione peroxidase
NapA	*trxR*	P	NAP-1	*cys-9*	P	Thioredoxin reductase
NapA	*gstA*	P	NAP-1	*gst-2*	P	Glutathione S-transferase
NapA	*catB*	P	NAP-1	*cat-3*	P	Catalase
NirA	*nrtB*	P	NIT-4	*nit-10*	P	High-affinity nitrate transporter
NirA	*niaD*	Both	NIT-4	*nit-3*	Both	Nitrate reductase
PacC	*palF*	P	PACC;PAC-3	*pal-6*	B	pH signaling protein
PacC	*pacC*	P	PACC;PAC-3	*pacc;pac-3*	B	Transcription factor for pH response
QutA	*qutA*	P	qa-1F	*qa-1f*	Both	Positive regulator of the quinic acid utilization cluster
QutA	*qutE*	B	qa-1F	*qa-2*	Both	3-Dehydroquinate dehydratase
QutR	*qutA*	P	qa-1S	*qa-1f*	P	Positive regulator of the quinic acid utilization cluster
QutR	*qutB*	P	qa-1S	*qa-3*	P	Quinate dehydrogenase
SreA	*sidA*	P	SRE	*ono*	P	Ornithine N5-monooxygenase
SreA	*catB*	P	SRE	*cat-3*	P	Catalase
UaY	*hxA*	Both	PCO1	*xdh-1*	Both	Xanthine dehydrogenase
XlnR	*xkiA*	P	XLR-1	*xyk-1*	P	D-xylulokinase
XlnR	*xdhA*	P	XLR-1	NCU00891	P	Xylitol dehydrogenase
XlnR	*xlnB*	P	XLR-1	*gh11-2*	P	Endo-1,4-beta-xylanase

a *B, binding experiments; P, TF-perturbation experiments; Both, both the above two types of experiments. Only low-throughput experiments were considered here*.

b *Functional annotations in A. nidulans*.

In addition, some regulatory interactions supported only by high-throughput experiments in one species were corroborated by low-throughput experiments on putative orthologous TFs and targets in the other species. These include 20 and 6 interactions (supported only by high-throughput experiments) in *A. nidulans* and *N. crassa*, respectively (Table S3). As an example, regulation of clock-controlled gene *ccg-1* by ATF-1 was supported by EMSA and Northern blot in *N. crassa* (Yamashita et al., 2008), and the orthologous regulation was also reported in *A. nidulans* through microarray analysis of wild-type and TF-deletion mutant (Emri et al., 2015), suggesting conserved regulatory mechanisms between the species.

Most of the transcriptional regulatory interactions curated in this study were only described in one of the two species but not in the other, which could be due to the research bias in the selection of TFs and targets, and the quick evolution of regulatory networks. Considering that *A. nidulans* and *N. crassa* belonging to Eurotiomycetes and Sordariomycetes, respectively, diverged about 300 million years ago (Hyde et al., 2017), it is not surprising that only a small fraction of regulatory interactions were identified as conserved between the two species. We then looked for the orthologs of TFs and targets involved in the above-mentioned 66 regulatory interactions (supported by low-throughput experiments in at least one of the two species) in the more distant *S. cerevisiae*. Twelve of the interactions also existed in *S. cerevisiae* according to the records in YEASTRACT database (Teixeira et al., 2014; Table S3). For example, *A. nidulans* AcuM, *N. crassa* AOD-2, and *S. cerevisiae* Rds2, which are orthologous proteins, all regulate genes in gluconeogenesis. While the regulation of *PCK1* and *FBP1* by Rds2 in *S. cerevisiae* is conserved in *A. nidulans* and *N. crassa*, AcuM/AOD-2 expand their functions to the control of alternative oxidase gene *aodA*/*AOD-1*, which is not present in *S. cerevisiae*. For Rim101 (Peñalva et al., 2008) and Gcn4 (Hoffmann et al., 2001) as pH and amino acid starvation responsive regulators, respectively, in *S. cerevisiae*, their auto-regulation was detected in all three species. On the other hand, this comparison indicated remarkable rearrangement of regulatory networks during evolution (Table S3). For example, although *XKS1* and *GRE1* involved in xylose metabolism in *S. cerevisiae* have their orthologs in *A. nidulans* and *N. crassa*, their regulators in the latter two species (XlnR/XLR-1) could not be found in *S. cerevisiae*. Taken together, the results suggested that a conserved TF could switch its regulon, and a new TF could arise to regulate conserved genes, during the evolution of fungi (Lavoie et al., 2009; Vaquerizas et al., 2009). The possible evolutionary events resulting in the diverse transcriptional regulatory networks are expected to be elucidated by comparing the regulatory interactions in more fungal species.

## Conclusion

In this study, transcriptional regulatory interactions described in *A. nidulans* and *N. crassa* were comprehensively collected and curated, providing a catalog of the studied regulatory relationships. Network motifs of different structures were identified by analyzing the reconstructed transcriptional regulatory networks, and a small proportion of the regulatory interactions were found to be conserved between the two species. The curated data are machine readable, and therefore can be easily incorporated to the already developed databases (e.g., AspGD). Considering the data collected here only represent a partial picture of the whole transcriptional regulatory network, experimental studies (particularly high-throughput ones) on more TFs are expected to be performed to provide more information about their regulons. The workflow and criteria used in this study could be references for updating the current data in the future. Also, other types of data related to transcriptional regulation (e.g., consensus TF-binding motifs) need to be curated. Finally, collection of transcriptional regulatory interactions in other important filamentous fungal species would be valuable for both application and evolution studies.

## Author contributions

YH and YQ: curated the regulatory interactions and revised the manuscript; GL: curated the regulatory interactions, analyzed the data, and drafted the manuscript.

### Conflict of interest statement

The authors declare that the research was conducted in the absence of any commercial or financial relationships that could be construed as a potential conflict of interest. The reviewer BF and handling Editor declared their shared affiliation.

## Supplementary material

Figure S1 Distribution of the number of experimental evidences per TF–target interactions. The interaction with most evidences in each species is shown.

Figure S2 Visualized transcriptional regulatory networks of high resolution in *A. nidulans* **(A)** and *N. crassa* **(B)**.

Figure S3 Regulatory interactions between TFs involved in conidiation in *A. nidulans*. All the interactions are supported by low-throughput experiments. The nodes and arrows are styled as indicated in the legend of Figure 2.

Table S1 Curated transcriptional regulatory interactions in *A. nidulans* and *N. crassa*.

Table S2 Autoregulating transcription factors and transcription factor cascades.

Table S3 Orthologous regulatory interactions evidenced in both *A. nidulans* and *N. crassa*.
