# Corrigendum: Collection and Curation of Transcriptional Regulatory Interactions in *Aspergillus nidulans* and *Neurospora crassa* Reveal Structural and Evolutionary Features of the Regulatory Networks

**DOI:** 10.3389/fmicb.2018.02713

**Published:** 2018-11-08

**Authors:** Yibo Hu, Yuqi Qin, Guodong Liu

**Affiliations:** ^1^State Key Laboratory of Microbial Technology, School of Life Science, Shandong University, Jinan, China; ^2^Hunan Provincial Key Laboratory of Microbial Molecular Biology, State Key Laboratory of Developmental Biology of Freshwater Fish, College of Life Science, Hunan Normal University, Changsha, China; ^3^National Glycoengineering Research Center, Shandong University, Jinan, China

**Keywords:** transcription factor, transcriptional regulatory network, filamentous fungi, *Aspergillus nidulans*, *Neurospora crassa*

In the original article, there was an error. A reference paper evidencing the effect of autoregulation of transcription factor AlcR in *Aspergillus nidulans* (Lockington et al., 1987) was missed during data collection. Thus, the description that “the consequent regulatory effects have not been clarified through low-throughput experiments” is incorrect for AlcR. The citation has been added and a correction has made to Results and Discussion, Structural Features of Transcriptional Regulatory Networks, Autoregulation:

Twelve and eight TFs were identified as autoregulating TFs in *A. nidulans* and *N. crassa*, respectively (Table S2). Specifically, in *A. nidulans*, AlcR (Lockington et al., 1987), AbaA, BrlA, QutA, PacC, StuA, and AreA activate, while QutR, CreA, and HapB repress, their own expression. Also, WC-1, CPC-1, ACR-2, QA-1F, and FL activate their own expression in *N. crassa*. These autoregulations might enhance or attenuate the regulatory outputs of TFs in response to environmental changes. In addition, VosA and CpcA in *A. nidulans* and PACC, CYS-3, SRE in *N. crassa* can bind to their own promoters, while the consequent regulatory effects have not been clarified through low-throughput experiments to our knowledge.

The authors apologize for this error and state that this does not change the scientific conclusions of the article in any way. The original article has been updated.

## Conflict of interest statement

The authors declare that the research was conducted in the absence of any commercial or financial relationships that could be construed as a potential conflict of interest.
