# Supplementary figures and images for: Collection and Curation of Transcriptional Regulatory Interactions in Aspergillus nidulans and Neurospora crassa Reveal Structural and Evolutionary Features of the Regulatory Networks
Source: Front Microbiol. 2018 Jan 19;9:27. doi: 10.3389/fmicb.2018.00027 (PMC5780447; doi:10.3389/fmicb.2018.00027)

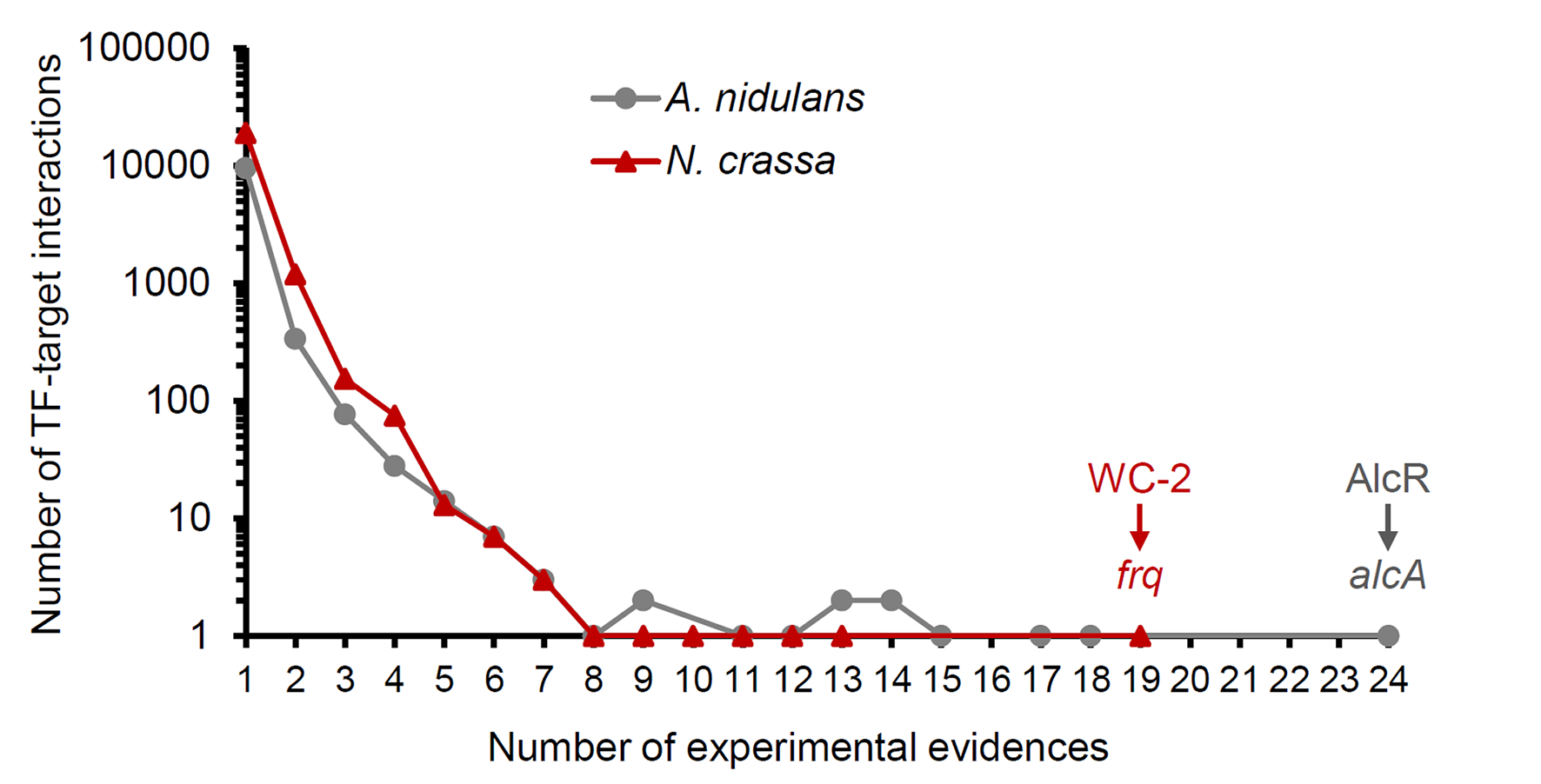

Supplement: Figure S1 — Distribution of the number of experimental evidences per TF–target interactions. The interaction with most evidences in each species is shown. [file Image1.TIF]

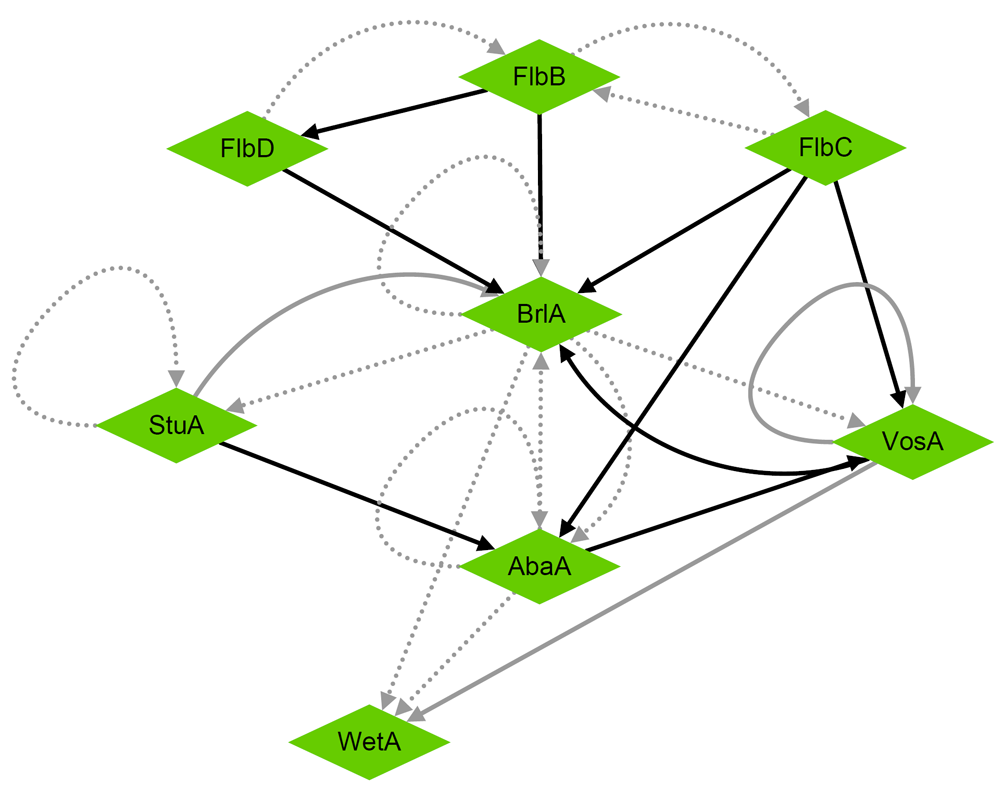

Supplement: Figure S3 — Regulatory interactions between TFs involved in conidiation in A. nidulans. All the interactions are supported by low-throughput experiments. The nodes and arrows are styled as indicated in the legend of Figure 2. [file Image3.TIF]
