# Supplementary material for: Collection and Curation of Transcriptional Regulatory Interactions in Aspergillus nidulans and Neurospora crassa Reveal Structural and Evolutionary Features of the Regulatory Networks
Source: Front Microbiol. 2018 Jan 19;9:27. doi: 10.3389/fmicb.2018.00027 (PMC5780447; doi:10.3389/fmicb.2018.00027)
Supplement: Figure S2 — Visualized transcriptional regulatory networks of high resolution in A. nidulans (A) and N. crassa (B). [file Image2.PDF]

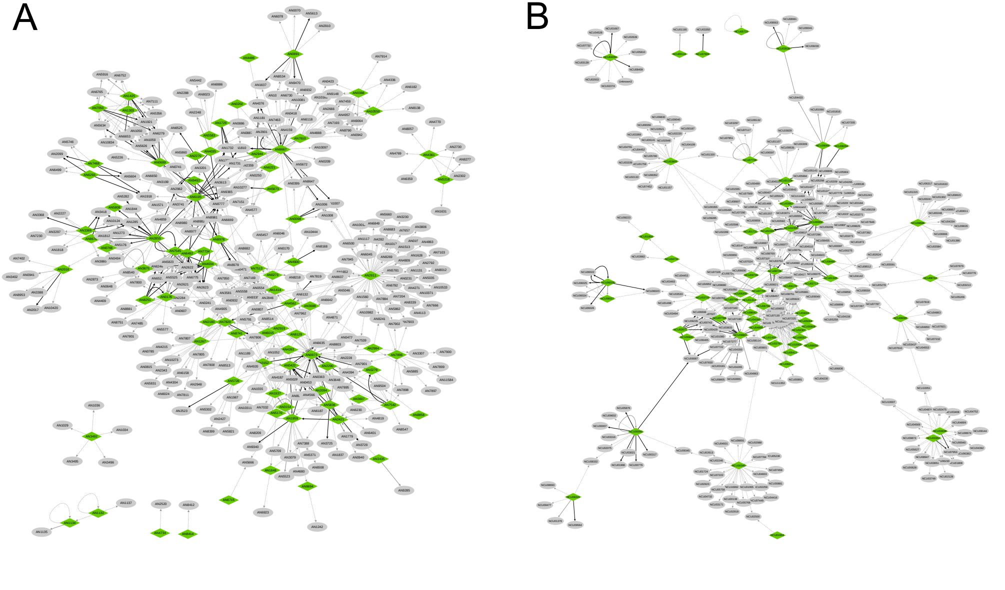

Figure S2 Visualized transcriptional regulatory networks of high resolution in *A. nidulans* (A) and *N. crassa* **(B)**.
